# Mechanisms Linking Oxidative Stress and Sarcopenia in Cardiovascular Diseases: A Scoping Review

**DOI:** 10.3390/antiox15020184

**Published:** 2026-02-01

**Authors:** Sabina Krupa-Nurcek, Tomasz Semań, Mateusz Szczupak, Jacek Kobak, Wioletta Mędrzycka-Dąbrowska, Kazimierz Widenka

**Affiliations:** 1 Department of Surgery, Faculty of Medicine, Collegium Medicum, University of Rzeszów, 35-310 Rzeszów, Poland; sabinakrupa@o2.pl (S.K.-N.); tseman@ur.edu.pl (T.S.); kwidenka@ur.edu.pl (K.W.); 2 Department of Ansesthesiology and Intensive Therapy, Copernicus Hospital, 80-803 Gdańsk, Poland; szczupak.mateusz@icloud.com; 3 Department of Otolaryngology, Faculty of Medicine, Medical University of Gdańsk, 80-210 Gdańsk, Poland; 4 Department of Anaesthesiology Nursing & Intensive Care, Faculty of Health Sciences, Medical University of Gdansk, 80-211 Gdańsk, Poland; wioletta.medrzycka-dabrowska@gumed.edu.pl

**Keywords:** oxidative stress, sarcopenia, cardiovascular diseases, scoping review

## Abstract

Oxidative stress and sarcopenia are increasingly perceived as interdependent processes that significantly affect the course of cardiovascular diseases. Excessive production of reactive oxygen species leads to muscle cell damage, mitochondrial disorders, and chronic inflammation, which promote progressive loss of muscle mass and function. Methods: The aim of the study was to analyze the mechanisms linking oxidative stress and sarcopenia in the course of cardiovascular diseases. Our scoping review initially identified 854 articles, of which 3 were ultimately included in the review (after removing duplicates (n = 118), 736 articles remained; after re-screening the articles according to the inclusion and exclusion criteria (n = 302), 434 articles remained; 196 publications lacked full text and were excluded, leaving 238 articles). Results: An examination of the available literature indicates a potential association between increased oxidative stress and the possible development of sarcopenia in individuals with cardiovascular diseases. The studies identified in this review suggest that elevated levels of reactive oxygen species, together with reduced antioxidant capacity, may contribute to muscle fiber damage, mitochondrial disturbances, and the activation of chronic inflammatory processes, which could in turn be involved in the accelerated decline of muscle mass and strength. Conclusions: These results confirm that oxidative stress is a key pathophysiological element linking both disease entities and may be an important target of therapeutic interventions.

## 1. Introduction

Sarcopenia, or progressive loss of muscle mass and function, is closely linked to an increase in oxidative stress. According to scoping reviews, mitochondria, which are the main source of reactive oxygen species (ROS), are disrupted [1,2,3,4]. Damaged mitochondria produce more ROS, while at the same time, cells lose their ability to effectively detoxify free radicals, as the activity of antioxidant enzymes such as superoxide dismutase (SOD), catalase or glutathione peroxidase decreases [5,6]. At the biochemical level, an excess of reactive oxygen species initiates a self-perpetuating process that results in oxidative modifications to muscle proteins, peroxidation of membrane lipids, and damage to mitochondrial DNA [6,7,8]. These alterations further aggravate mitochondrial dysfunction, contributing to a reinforcing cycle of rising oxidative stress and progressive muscle loss [9,10,11]. Additionally, the chronic inflammation associated with sarcopenia increases the activity of the NF-κB and MAPK pathways, which further stimulates ROS production and accelerates muscle protein degradation by the ubiquitin–proteasome system [12].

Sarcopenia is associated with profound metabolic disorders that intensify oxidative stress and are simultaneously exacerbated by it [13]. Mitochondrial dysfunction plays a key role—in conditions of muscle mass deficiency, the respiratory chain, especially complex I and III, is weakened, which promotes the “escape” of electrons and the excessive production of reactive oxygen species [13,14,15,16]. ROS activate protein degradation pathways, the ubiquitin–proteasome system and autophagy, as well as increase the activity of MAPK kinases and the transcription factor NF-κB, which are responsible for chronic inflammation and further loss of muscle fibers. At the same time, the antioxidant system, including the key glutathione pathway, is weakened [17,18]. Glutathione (GSH) is the primary reducer in the cell, and its regeneration depends on the activity of glutathione dehydrogenase and the availability of NADPH, produced in the pentose phosphate pathway [19]. In sarcopenia, a decrease in the GSH/GSSG ratio is observed, which indicates an overload of the antioxidant system and increased susceptibility of proteins and lipids to oxidative damage [20]. Diet and physical activity are of fundamental importance here: regular aerobic and resistance exercise improves mitochondrial biogenesis by activating PGC-1α, increases the activity of antioxidant enzymes, and increases the ability of muscles to regenerate glutathione [19,20,21]. On the other hand, a diet rich in antioxidants—such as polyphenols and vitamins C and E, as well as sulfur amino acids, which are precursors of glutathione—supports the maintenance of the oxidative and antioxidant balance. As a result, proper nutrition and exercise can break the vicious cycle of oxidative stress and muscle degradation, slowing the progression of sarcopenia and improving the body’s metabolic function [20,21,22].

## 2. Materials and Methods

### 2.1. Study Design

We adopted a scoping review methodology because our aim was to develop a conceptual map illustrating the mechanisms linking oxidative stress and sarcopenia in cardiovascular disease. Scoping reviews represent a relatively recent research approach, and there is still a lack of clear guidance on when to select a systematic review versus a scoping review, particularly in situations where the available literature is insufficiently developed, highly extensive, complex, or heterogeneous, making a more detailed systematic review unfeasible [23]. Our scoping review was conducted in accordance with the methodological framework outlined in the Joanna Briggs Institute’s manual for scoping reviews and followed the recommendations of the PRISMA-ScR (Preferred Reporting Items for Systematic Reviews and Meta-Analyses for Scoping Reviews) guidelines [24,25].

### 2.2. Inclusion and Exclusion Criteria

To capture the essential elements of the mechanisms connecting oxidative stress with sarcopenia in cardiovascular disease, we developed a research question that clearly specified the target population, the central concept, and the contextual framework of the review.

The inclusion criteria were: articles published between 2015 and 2025; original research (observational studies and randomized trials), meta-analyses, systematic reviews, and narrative reviews; studies with full-text availability; publications written in English.

The exclusion criteria were: case reports, commentaries, letters to the editor, and book chapters; studies without accessible full text; publications written in languages other than English.

*Population*

The review encompassed studies addressing sarcopenia and oxidative stress in individuals diagnosed with cardiovascular diseases. In this context, sarcopenia was defined as a progressive condition marked by age-related declines in muscle mass, strength, and functional capacity. Oxidative stress was understood as a state of imbalance in which excessive levels of unstable molecules—free radicals—overwhelm the body’s antioxidant defenses, leading to cellular, protein, and DNA damage and contributing to aging as well as various diseases, including cancer, cardiovascular disorders, and neurodegenerative conditions [3,5,11,19].

*Concept*

The focus was on sarcopenia and oxidative stress in patients with cardiovascular disease. The aim of the study was to analyze the mechanisms linking oxidative stress and sarcopenia in the course of cardiovascular diseases.

*Context*

The studies to be included in the review included patients with cardiovascular diseases.

### 2.3. Search Strategy

Three authors conducted a comprehensive search across the following databases: PubMed, Scopus, EBSCO, Web of Science and the Cochrane Library. The keywords applied included: “sarcopenia,” “oxidative stress,” “cardiovascular diseases,” “sarcopenia and oxidative stress,” “sarcopenia in cardiovascular diseases,” and “oxidative stress in cardiovascular diseases.” A substantial number of studies focused exclusively on the biochemical aspects of sarcopenia and oxidative stress, which did not meet the scope of this review. The authors also identified many publications addressing conditions unrelated to cardiovascular disease. Keywords and their combinations were entered using the AND and OR operators. All retrieved publications were screened by title and abstract to eliminate irrelevant records. Any disagreements were resolved through discussion among five investigators, and full consensus was ultimately achieved regarding the final set of included articles. The initial search was conducted between 11 November 2025, and 20 December 2025.

### 2.4. Extraction of Data

A data extraction form developed in accordance with the JBI guidelines for scoping reviews [24] was applied to capture key information from the included studies. The extraction procedure—referred to in scoping reviews as “data plotting” [26]—was conducted independently by two reviewers. The Population–Concept–Context (PCC) framework guided the identification of relevant publications. For each study, information such as the first author, year of publication, country, study design, study aim, inclusion and exclusion criteria (PCC), results, and principal conclusions was collected. All data were organized and managed using Microsoft Excel.

### 2.5. Critical Appraisal Process

A scoping review may include a review of current evidence without including a methodological assessment of the included studies [24].

### 2.6. Process for Including Publications to the Review

**Figure 1:**
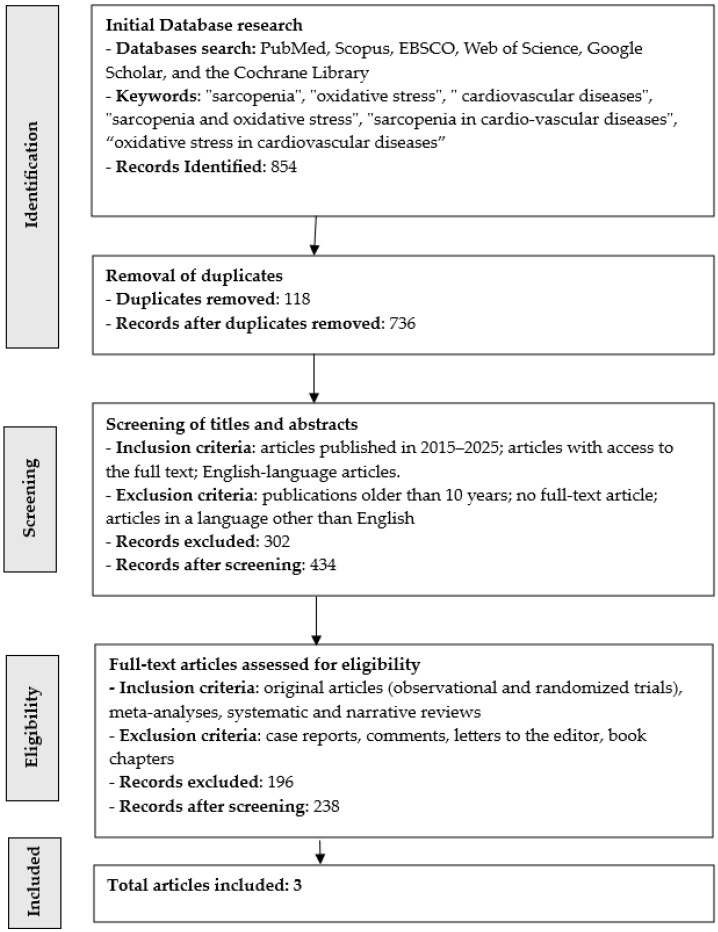
Literature search and selection flowchart for this review—PRISMA-ScR.

Our scoping review initially identified 854 records, of which 3 were ultimately included (Figure 1). After removing duplicates (n = 118), 736 articles remained. A subsequent screening based on the predefined inclusion and exclusion criteria eliminated 302 records, leaving 434 articles. Of these, 196 lacked full-text availability and were excluded, resulting in 238 articles eligible for further assessment. Ultimately, 3 studies met all criteria and were included in the final review. The drastic reduction of studies to 3 was associated with a lack of full texts and a lack of papers in English, and there were also papers that focused on the biochemical elements of sarcopenia and oxidative stress (not only in cardiology). These studies were conducted in Italy (n = 2) and Hungary (n = 1). The findings are summarized in Table 1.

## 3. Sarcopenia as a Catalyst for Oxidative Stress

In recent years, there has been increasing attention paid to the role of oxidative stress as a key element of the pathophysiology of sarcopenia. Although oxidative stress may be a factor initiating the process of muscle degradation, as the disease progresses, sarcopenia itself begins to further fuel the formation of reactive oxygen species (ROS), creating a vicious circle of worsening muscle dysfunction [18,30]. Understanding this mechanism is clinically important because it allows for a better assessment of the risk of complications in elderly, chronically ill or immobilized patients, as well as identifying potential therapeutic targets. In patients with advanced sarcopenia, marked changes in the structure and function of muscle fibers are observed [12]. There is a reduction in the number of mitochondria and disorders of their biogenesis, which is associated with reduced activity of the PGC-1α transcriptional regulator. Mitochondria that remain in cells often show features of dysfunction: mitochondrial DNA damage, disorders in the composition of membrane phospholipids, and reduced efficiency of the respiratory chain. Under such conditions, there is an increased “escape” of electrons from complexes I and III, which leads to excessive production of superoxide anion radicals [9,14]. In healthy muscles, ROS play a signaling role, regulating exercise adaptation and regenerative processes, but in sarcopenia, their amount exceeds the ability of antioxidant systems to neutralize them. With the progressive loss of muscle mass, the activity of enzymes responsible for detoxifying free radicals is weakened. Reduced availability of NADPH, which is necessary for glutathione regeneration, is due to a weakening of the pentose phosphate pathway activity and dysfunction of enzymes such as glucose-6-phosphate dehydrogenase. As a result, muscle cells become more susceptible to oxidative damage to proteins, lipids and nucleic acids. At the clinical level, patients with sarcopenia often present symptoms that indirectly reflect increased oxidative stress [4,8,12]. Reduced muscle strength, rapid fatigue, slowing of gait or difficulties in performing daily activities result not only from the loss of muscle mass, but also from the deterioration of the functional quality of the fibers. Oxidative damage to contractile proteins such as actin and myosin leads to disruption in the generation of contraction force. Peroxidation of lipids of cell membranes affects the integrity of the sarcolemma, disrupting ionic conduction and increasing the susceptibility of cells to apoptosis. In turn, mitochondrial DNA damage limits the ability of cells to produce ATP effectively, which further exacerbates muscle fatigue [10,15,16,17].

As sarcopenia progresses, catabolic pathways are also activated, which themselves generate additional oxidative stress. The ubiquitin–proteasome system, responsible for the degradation of damaged proteins, is overactivated under the influence of ROS and pro-inflammatory cytokines such as TNF-α and IL-6. At the same time, intensified autophagy, although initially having a protective function, leads to the degradation of healthy cellular structures under chronic oxidative stress, which further weakens the muscles. Activation of the transcription factor NF-κB, which is a response to ROS, enhances inflammation and increases the expression of genes encoding catabolic proteins. In this way, sarcopenia is not only a consequence of oxidative stress, but also becomes its source [22,31].

It is worth noting that patients with chronic diseases such as heart failure, chronic obstructive pulmonary disease, type 2 diabetes or chronic kidney disease are particularly at risk of increased coupling between sarcopenia and oxidative stress. In these diseases, there is an increase in ROS production and metabolic disorders that accelerates the loss of muscle mass at an early stage. In turn, sarcopenia itself worsens the prognosis, increases the risk of hospitalization and reduces the quality of life [16,19]. In immobilized or long-term bedridden patients, there is an additional decrease in the activity of mitochondrial enzymes, which further intensifies oxidative stress and accelerates muscle degradation. The biochemical consequences of sarcopenia also have a systemic dimension. Skeletal muscles are the largest store of amino acids in the body, and their degradation leads to an increase in the concentration of free amino acids in the blood, which can disrupt metabolic balance and promote gluconeogenesis. An increase in ROS levels affects the functioning of other organs, including the liver and immune system, intensifying inflammatory processes and disrupting metabolic homeostasis. In this way, local changes in muscle tissue become part of systemic oxidative–inflammatory dysfunction [4,11,16].

Sarcopenia is not only a passive effect of oxidative stress, but also actively fuels it through mitochondrial dysfunction, weakening of antioxidant systems, activation of catabolic pathways and intensification of chronic inflammation. Understanding this bidirectional relationship is crucial for developing effective therapeutic strategies, which should include both nutritional and supplementation interventions and appropriately selected physical activity, aimed at improving mitochondrial function and increasing the body’s antioxidant potential. In clinical practice, this means the need for early diagnosis of sarcopenia and a comprehensive approach to patients who are at particularly high risk of oxidative disorders [21,32].

## 4. The Effect of Oxidative Stress on the Development of Sarcopenia

Oxidative stress is one of the key pathophysiological factors leading to the development of sarcopenia, i.e., progressive loss of muscle mass and function. With age or in the course of chronic diseases, there is an imbalance between the production of reactive oxygen species (ROS) and the body’s ability to neutralize them [7,14]. Excess ROS causes a number of biochemical and structural changes in muscle tissue, which gradually lead to weakening of fibers, their degradation and impairment of their regenerative abilities. Understanding the mechanisms by which oxidative stress affects muscles can better explain why sarcopenia develops so dynamically in the elderly, patients with metabolic diseases or immobilized people. One of the most important sites of ROS formation in muscle cells is mitochondria [18,33]. Under physiological conditions, a small amount of free radicals has a signaling function, regulating exercise adaptation, mitochondrial biogenesis and repair processes. However, with age or under the influence of stress factors, there is a dysfunction of the respiratory chain, especially complexes I and III, which leads to increased electron “escape” and overproduction of superoxide anion radicals. Damage to mitochondrial DNA, which does not have as effective repair mechanisms as nuclear DNA, further exacerbates mitochondrial dysfunction. As a result, a vicious circle is created: damaged mitochondria generate more ROS, and ROS exacerbate mitochondrial damage, leading to a gradual decline in the ability of muscle cells to produce energy [34].

Excessive ROS also affects the structure and function of muscle proteins. Reactive oxygen species can modify contractile proteins such as actin and myosin, leading to disruption in the generation of contraction force. Oxidative damage to enzymatic proteins disrupts the functioning of metabolic pathways, including glycolysis, the Krebs cycle or oxidative phosphorylation [6]. ROS can also cause protein aggregation, which hinders protein degradation and leads to the accumulation of abnormal structures in muscle cells. In response to these changes, catabolic pathways such as the ubiquitin–proteasome system and autophagy are activated, which aim to remove damaged proteins, but under conditions of chronic oxidative stress lead to excessive degradation of healthy cellular elements [9,14]. Another important mechanism is the effect of oxidative stress on cell membrane lipids. Lipid peroxidation leads to loss of sarcollegmic integrity, ionic conduction disorders and increased susceptibility of cells to apoptosis. Lipid peroxidation products such as 4-hydroxynonenal (4-HNE) have a toxic effect on muscle cells, modifying proteins and exacerbating inflammation. As a result, muscle fibers become more fragile and less resistant to mechanical and metabolic damage, which accelerates their degradation. Oxidative stress also affects the regulation of inflammatory processes, which play a key role in the development of sarcopenia. ROS activate the transcription factor NF-κB, which increases the expression of pro-inflammatory cytokines such as TNF-α, IL-1β or IL-6. These cytokines enhance muscle protein catabolism, inhibit protein synthesis by blocking the mTOR pathway, and increase proteasome activity [11,13,21,34]. Chronic inflammation, referred to as “inflammaging”, is one of the main factors accelerating the development of sarcopenia in the elderly. ROS can also activate MAPK kinases, which are involved in the regulation of apoptosis and stress response, which further exacerbates muscle degradation. An additional important element of the pathogenesis of sarcopenia is the weakening of antioxidant systems [3,7,15,19]. With age, the activity of enzymes such as superoxide dismutase, catalase or glutathione peroxidase decreases. Particularly important is the decrease in glutathione (GSH), which is the main cellular antioxidant. A decrease in the GSH/GSSG ratio indicates an overload of the reducing system and an increased susceptibility of cells to oxidative damage. Deficiency of NADPH, necessary for the regeneration of glutathione, results from weakening of the activity of the pentose phosphate pathway and dysfunction of enzymes responsible for maintaining the redox balance [31,32,33]. As a result, muscle cells lose their ability to neutralize ROS, which accelerates degenerative processes. Oxidative stress also affects satellite cells, which are responsible for muscle regeneration. ROS can inhibit their proliferation and differentiation, leading to a decrease in the repair capacity of muscle tissue. DNA damage to and disruption of Wnt and Notch signaling, which are crucial for satellite cell activation, further limit fiber regeneration. As a result, even minor muscle damage that would be quickly repaired in a young body leads to permanent muscle loss in the elderly [35,36].

In a clinical context, oxidative stress contributes to the deterioration of muscle function, decreased strength, slowed gait and increased risk of falls. Patients with chronic diseases, such as type 2 diabetes, heart failure, chronic obstructive pulmonary disease or chronic kidney disease, are particularly at risk of developing sarcopenia, because their body is characterized by an increased level of oxidative stress at the outset [37]. In these cases, ROS not only damages the muscles but also disrupts hormonal balance, including insulin and IGF-1 signaling, which further inhibits muscle protein synthesis. Oxidative stress plays a fundamental role in the development of sarcopenia through its multidirectional effects on mitochondria, proteins, lipids, DNA, inflammatory processes and antioxidant systems [9,12]. Excess ROS leads to metabolic dysfunctions, degradation of cellular structures and impaired muscle regeneration, which in the long term results in a progressive loss of muscle mass and strength. Understanding these mechanisms is crucial for developing effective therapeutic strategies, which should include both nutritional and supplementation interventions and regular physical activity to support mitochondrial function and oxidation-antioxidant balance [17,18,19,33,36].

## 5. Sarcopenia and Oxidative Stress in Patients with Heart Disease

The development of sarcopenia and the intensification of oxidative stress are two key interrelated pathophysiological processes that significantly affect the course of heart disease, prognosis and convalescence of cardiac and cardiac surgery patients [8]. In recent years, it has become increasingly clear that cardiovascular diseases are not limited to myocardial or vascular pathologies, but encompass a wide range of systemic changes, including metabolic, inflammatory and degenerative disorders [27]. Sarcopenia—defined as a progressive loss of muscle mass and strength—and oxidative stress—resulting from the excessive production of reactive oxygen species (ROS) while weakening antioxidant mechanisms—are now recognized as significant factors in the deterioration of the functioning of the body of a patient with heart disease [19,20,21,22].

The mechanisms leading to heart damage in the course of oxidative stress are multidimensional. Reactive oxygen species act directly on cellular structures, leading to membrane lipid peroxidation, protein damage and DNA fragmentation. In cardiomyocytes, this results in a disruption of mitochondrial function, which is crucial for energy production in the heart [11]. Mitochondrial dysfunction leads to a decrease in the ability of cells to generate ATP, which—in conditions of increased energy demand, typical of heart failure—aggravates systolic and diastolic insufficiency [14]. In addition, oxidative stress activates inflammatory pathways, including NF-κB, intensifying myocardial fibrosis processes. This fibrosis, in turn, reduces the flexibility and contractility of the heart, contributing to the progression of heart failure and an increased risk of arrhythmia [32,33,34].

Sarcopenia, although it primarily affects skeletal muscles, also has a significant impact on the cardiovascular system. Loss of muscle mass is associated with a decrease in overall physical performance, reduced metabolic reserves and greater susceptibility to complications [35]. Mechanisms leading to sarcopenia in patients with heart disease include chronic inflammation, hormonal disorders (including decrease in testosterone, growth hormone, and IGF-1 levels), malnutrition, as well as reduced physical activity resulting from disease restrictions. It is worth noting that oxidative stress and sarcopenia intensify each other—ROS accelerate the degradation of muscle proteins, and weakened muscles produce fewer myokines with anti-inflammatory and protective effects, which exacerbates metabolic dysfunction [34,35,36]. In the context of cardiac and cardiac surgery patients, both processes are of particular importance. In people with heart failure, sarcopenia is one of the most important predictors of a worse prognosis. Studies indicate that patients with reduced mass and muscle strength have a higher risk of hospitalization, more frequent exacerbations of the disease and increased mortality. This is due to the fact that skeletal muscles have not only a mechanical, but also a metabolic and immune function. Their weakening leads to a decrease in exercise tolerance, which limits the possibilities of cardiac rehabilitation—a key element of treatment after heart attack, cardiac surgery or heart failure. A patient with sarcopenia gets tired faster and recovers more slowly, and his body copes worse with hemodynamic loads [34,35,36,37].

Oxidative stress further complicates the course of heart disease, affecting the healing and regeneration processes. After cardiac surgery, such as coronary artery bypass grafting or valve replacement, the body experiences a significant increase in oxidative stress associated with tissue reperfusion, the use of extracorporeal circulation and an inflammatory response. Excess ROS can delay wound healing, increase the risk of infectious complications, and impair organ function, including the kidneys and lungs [28,38]. In patients with pre-existing myocardial dysfunction, oxidative stress may lead to further deterioration of ejection fraction and an increased risk of ventricular arrhythmias. The co-occurrence of sarcopenia and oxidative stress creates a vicious circle that significantly hinders recovery. Reduced muscle strength limits their ability to cope with the metabolism of glucose and fatty acids, which leads to insulin resistance and further intensification of inflammatory processes [11,31]. In turn, chronic inflammation and oxidative stress accelerate the degradation of muscle proteins, exacerbating sarcopenia. As a result, a patient after heart surgery or an episode of acute coronary syndrome may experience a much slower recovery, greater susceptibility to complications and a poorer quality of life. It is also worth noting the impact of these processes on patient survival [24,31]. Both sarcopenia and high levels of oxidative stress are independent risk factors for increased mortality in heart disease. In patients with heart failure, the presence of sarcopenia is associated with a significantly worse prognosis, regardless of age, ejection fraction or concomitant diseases. Similarly, oxidative stress is associated with atherosclerosis progression, atherosclerotic plaque destabilization, and a higher risk of cardiovascular events such as heart attack or stroke [38].

Sarcopenia and oxidative stress are closely linked in patients with heart disease, creating a vicious circle that worsens both skeletal and cardiovascular function [31,33]. In heart disease, chronic inflammation and increased production of reactive oxygen species (ROS) occur, leading to damage to proteins, lipids, and mitochondria in muscle cells. This damage disrupts the functioning of mitochondria, which are crucial for energy production in muscles, and their dysfunction is one of the main mechanisms of sarcopenia development [34]. Excess ROS also intensifies muscle protein degradation by activating the ubiquitin–proteasome system and autophagy pathways, leading to loss of muscle mass and strength. At the same time, sarcopenia itself can exacerbate oxidative stress. Reduced muscle mass and impaired mitochondrial function limit the body’s ability to neutralize free radicals, which promotes further tissue damage. In patients with heart disease, muscle perfusion disorders also occur, which intensifies hypoxia and secondary production of ROS. Chronic inflammation, typical of heart failure and other cardiac conditions, further enhances this process, leading to progressive muscle loss and deterioration of physical performance [29]. As a result, oxidative stress and sarcopenia are mutually reinforcing, and their co-occurrence in cardiac patients is associated with a worse prognosis, a higher risk of hospitalization and reduced quality of life. Understanding these mechanisms is crucial for developing therapeutic strategies aimed at both muscle protection and oxidative stress reduction [37,38].

In summary, current evidence suggests that the development of sarcopenia and the severity of oxidative stress may represent important components of the pathophysiology of heart disease, although further research is needed to clarify the strength and consistency of these associations [39]. Both processes affect the functioning of the heart muscle, the overall efficiency of the body, the possibility of rehabilitation, and the prognosis of patients. Their co-occurrence leads to worsening survival, increased risk of complications and prolonged recovery after cardiac and cardiac surgery [40,41,42]. Understanding these relationships is crucial for a comprehensive approach to a patient with heart disease, including not only pharmacological and surgical treatment, but also assessment of nutritional status, muscle mass and oxidation–antioxidant balance [16,30,31,32].

**Figure 2:**
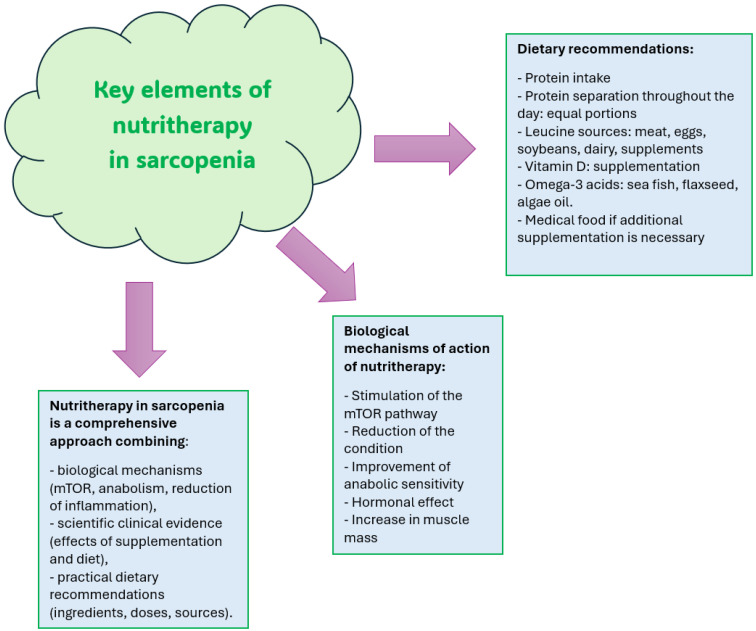
Key elements of nutritherapy used in sarcopenia.

It is also worth mentioning oxysterols, because as is known, some of these molecules are formed as a result of the autooxidation of cholesterol only when oxidative stress is strong. In addition, mitochondria are closely related to peroxisomes [43], and peroxisome dysfunctions are known to promote oxidative stress. Moreover, in vitro studies confirm that oxysterols promote mitochondrial and peroxisome dysfunctions and significant disruption of oxidative stress. These are the main points that open up new perspectives in the pathophysiology of sarcopenia and that should be presented in the next stages of research [44]. Figure 2 illustrates key elements of nutritherapy used in sarcopenia.

## 6. Conclusions

Current evidence suggests that sarcopenia—characterized by a progressive decline in muscle mass and strength—may be associated with an increased risk of cardiovascular disease, while oxidative stress is considered one of the potential biological mechanisms linking these conditions [45,46]. Excessive production of reactive oxygen species leads to muscle cell damage, mitochondrial disorders and chronic inflammation, which accelerates the degradation of muscle fibers and impairs the functioning of the circulatory system. In cardiovascular disease, oxidative stress exacerbates endothelial dysfunction, reduces muscle perfusion and promotes protein catabolism, creating a vicious circle between circulatory failure and muscle loss. In vitro studies support that peroxisomal damage could also occur as a consequence of mitochondrial damage [47]. A clear understanding of these mechanisms is essential for guiding the development of therapeutic approaches that have the potential to enhance both muscle function and cardiovascular health [17,48]. There are recent data in patients with sarcopenia supporting that the oxidative stress observed in patients with sarcopenia is strong oxidative stress. Indeed, increased levels of 7-ketocholesterol and 7-beta-hydroxycholesterol are found in the plasma of sarcopenic patients, and it is known that cholesterol is oxidized in oxisterols only when oxidative stress is strong. There are also many other arguments supporting oxidative stress, such as modulation of anti-oxidant enzyme activities, carbonylated proteins [47,49]. Oxysterols are considered biomarkers of oxidative stress. Future studies should address the potential role of peroxisomes in oxidative stress, as mitochondrial dysfunction may be mediated by mitochondrial dysfunction [50,51,52].

## 7. Limitations

This scoping review has several important limitations. The most significant is the very small number of studies that met the inclusion criteria, which limits the breadth of available evidence and reduces the ability to draw firm conclusions about the mechanisms linking oxidative stress and sarcopenia in cardiovascular diseases. Many potentially relevant publications were excluded due to lack of full-text availability or because they focused solely on biochemical aspects without addressing cardiovascular populations. In addition, no formal assessment of methodological quality was performed, in accordance with scoping review methodology, which means that the strength and reliability of the included evidence could not be critically evaluated. As a result, the findings should be interpreted with caution, and further well-designed studies are needed to clarify the nature and direction of the observed associations.

## Figures and Tables

**Table 1:** Characteristics and findings of studies included in this review.

Author, Year	Country	Participants	Findings
Bellanti F. et al., 2018 [27]	Italy	Patients with cardiovascular diseases	✓ Markers of oxidative stress are increased in sarcopenia, ✓ Oxidative stress is associated with cardiovascular disease risk in sarcopenic obesity ✓ Oxidative stress may be a pathophysiological link between sarcopenia and obesity
Sárközy M. et al., 2018 [28]	Hungary	Patients with kidney and cardiovascular diseases	✓ Mechanisms of oxidative stress could be involved in a complex response among the kidneys, heart and skeletal muscles ✓ Elevated oxidative/nitrosative stress and inflammation are key components of the complex pathomechanism and interrelationships between T4CRS and sarcopenia ✓ Understanding the mechanisms that lead to disorders in oxidative stress and their relationship to pro-inflammatory, hypertrophic, fibrotic, cellular, and other pathways would help develop strategies to counteract systemic and tissue-specific oxidative/nitrate stress to control the progression of kidney disease and heart damage
Armentaro G. et al., 2025 [29]	Italy	Elderly patients with chronic heart failure	✓ Addition of SGLT2i to therapy leads to improved echocardiographic and sarcopenia-related parameters and biomarkers of oxidative stress and platelet activation ✓ Inflammation and oxidative stress are two interrelated processes that play an important role in the development of heart failure

## Data Availability

No new data were created or analyzed in this study. Data sharing is not applicable to this article.

## Abbreviations

The following abbreviations are used in this manuscript:

ROS	reactive oxygen species
SOD	superoxide dismutase
DNA	deoxyribonucleic acid
NF-κB	nuclear factor kappa-light-chain-enhancer of activated B cells
MAPK	mitogen-activated protein kinases
GSH	glutathione
NADPH	nicotinamide adenine dinucleotide phosphate
GSSG	glutathione disulfide
PGC-1α	peroxisome proliferator-activated receptor gamma coactivator 1-alpha
PCC	Population–Concept–Context
GPx	glutathione peroxidase
ATP	adenosine triphosphate
TNF-α	tumour necrosis factor alpha
IL-6	interleukin 6
4-HNE	4-hydroxynonenal
